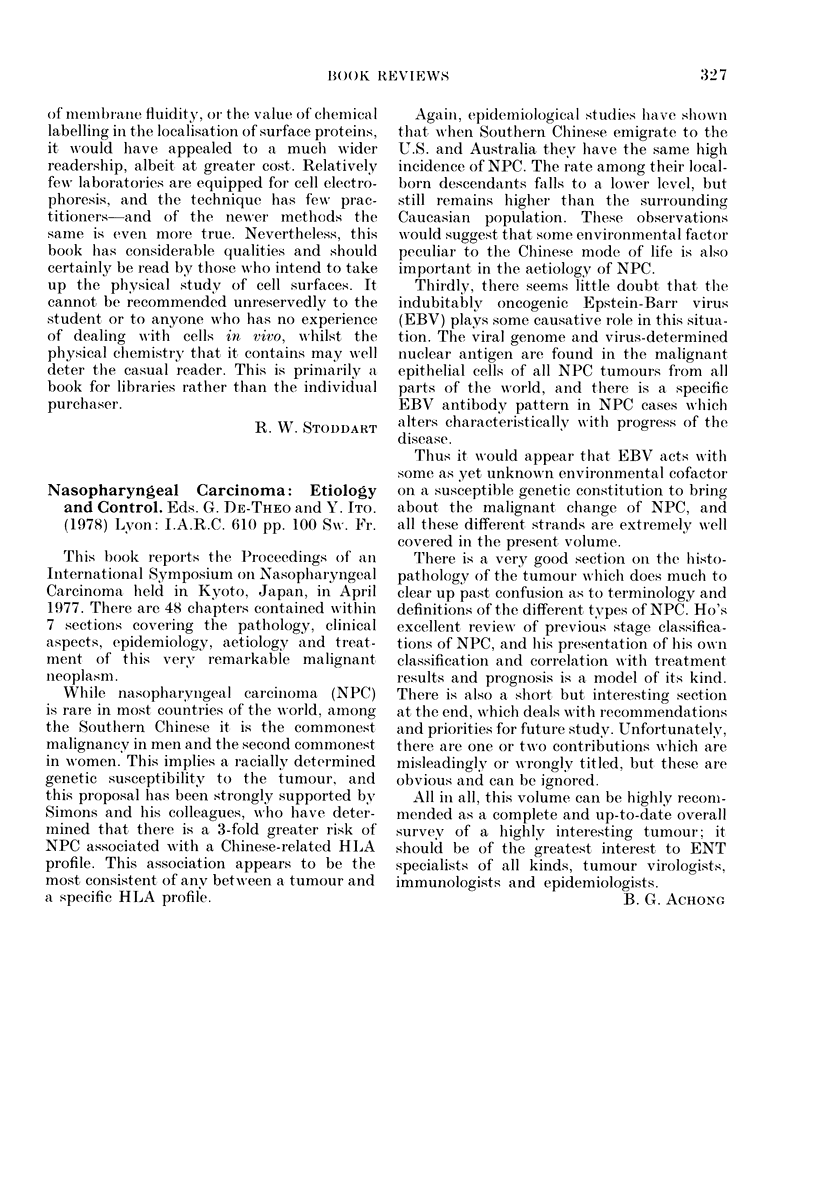# Nasopharyngeal Carcinoma: Etiology and Control

**Published:** 1979-08

**Authors:** B. G. Achong


**Nasopharyngeal Carcinoma: Etiology and Control.** Eds. G. DE-THEO and Y. ITO. (1978) Lyon: I.A.R.C. 610 pp. 100 Sw. Fr.

This book reports the Proceedings of an International Symposium on Nasopharyngeal Carcinoma held in Kyoto, Japan, in April 1977. There are 48 chapters contained within 7 sections covering the pathology, clinical aspects, epidemiology, aetiology and treatment of this very remarkable malignant neoplasm.

While nasopharyngeal carcinoma (NPC) is rare in most countries of the world, among the Southern Chinese it is the commonest malignancy in men and the second commonest in women. This implies a racially determined genetic susceptibility to the tumour, and this proposal has been strongly supported by Simons and his colleagues, who have determined that there is a 3-fold greater risk of NPC associated with a Chinese-related HLA profile. This association appears to be the most consistent of any between a tumour and a specific HLA profile.

Again, epidemiological studies have shown that when Southern Chinese emigrate to the U.S. and Australia they have the same high incidence of NPC. The rate among their local-born descendants falls to a lower level, but still remains higher than the surrounding Caucasian population. These observations would suggest that some environmental factor peculiar to the Chinese mode of life is also important in the aetiology of NPC.

Thirdly, there seems little doubt that the indubitably oncogenic Epstein-Barr virus (EBV) plays some causative role in this situation. The viral genome and virus-determined nuclear antigen are found in the malignant epithelial cells of all NPC tumours from all parts of the world, and there is a specific EBV antibody pattern in NPC cases which alters characteristically with progress of the disease.

Thus it would appear that EBV acts with some as yet unknown environmental cofactor on a susceptible genetic constitution to bring about the malignant change of NPC, and all these different strands are extremely well covered in the present volume.

There is a very good section on the histopathology of the tumour which does much to clear up past confusion as to terminology and definitions of the different types of NPC. Ho's excellent review of previous stage classifications of NPC, and his presentation of his own classification and correlation with treatment results and prognosis is a model of its kind. There is also a short but interesting section at the end, which deals with recommendations and priorities for future study. Unfortunately, there are one or two contributions which are misleadingly or wrongly titled, but these are obvious and can be ignored.

All in all, this volume can be highly recommended as a complete and up-to-date overall survey of a highly interesting tumour; it should be of the greatest interest to ENT specialists of all kinds, tumour virologists, immunologists and epidemiologists.

B. G. ACHONG